# Relativistic-microwave theory of ball lightning

**DOI:** 10.1038/srep28263

**Published:** 2016-06-22

**Authors:** H.-C. Wu

**Affiliations:** 1 Institute for Fusion Theory and Simulation (IFTS) and Department of Physics, Zhejiang University, Hangzhou 310027, China; 2 IFSA Collaborative Innovation Center, Shanghai Jiao Tong University, Shanghai 200240, China

## Abstract

Ball lightning, a fireball sometimes observed during lightnings, has remained unexplained. Here we present a comprehensive theory for the phenomenon: At the tip of a lightning stroke reaching the ground, a relativistic electron bunch can be produced, which in turn excites intense microwave radiation. The latter ionizes the local air and the radiation pressure evacuates the resulting plasma, forming a spherical plasma bubble that stably traps the radiation. This mechanism is verified by particle simulations. The many known properties of ball lightning, such as the occurrence site, relation to the lightning channels, appearance in aircraft, its shape, size, sound, spark, spectrum, motion, as well as the resulting injuries and damages, are also explained. Our theory suggests that ball lighting can be created in the laboratory or triggered during thunderstorms. Our results should be useful for lightning protection and aviation safety, as well as stimulate research interest in the relativistic regime of microwave physics.

Since Arago^1^ first extensively discussed ball lightning in 1838, this rare natural phenomenon still remains a riddle. Ball lightning^2^,^3^,^4^,^5^ exhibits very diverse characteristics, such as close association with ordinary lightning, globate structure with steady glow for 1–5 seconds, and mostly horizontal motion. Ball lightning can be formed even inside aircraft and closed rooms, permeate glass plates, decay explosively or silently, and produce sound and acrid odours. Many models of ball lightning have been proposed, but none have been fully accepted^6^. In particular, these theories do not explain the inscrutable appearance of ball lightning inside fully-screened aircraft^7^. Here, we propose a theory for ball lightning formation, which can explain its appearing in aircraft and many other properties.

Lodge^8^ considered that ball lightning might be excited by a standing electrical wave from lightning. Kapitza^9^ argued that ball lightning could be formed through air ionization at antinodes of electromagnetic standing waves in the microwave regime. Dawson and Jones^10^ proposed that ball lightning could be a microwave bubble confined inside a globate plasma shell. Continuous air ionization by the trapped microwave maintains the plasma shell^11^. By dimensional analysis, we have pointed out^12^ that a microwave bubble can be formed similarly as light solitons observed in the laser-plasma interaction. Such a microwave bubble contains a half-cycle standing-wave mode and is sketched in Fig. 1a. The microwave-type model of ball lightning can explain its permeation through glass plates. However, the origin of microwave emission from lightning was never found.

In this article, we propose a mechanism for microwave generation from lightning. As shown in Fig. 1b, we assume that in a ball lightning event a relativistic electron bunch is generated by lightning. When this bunch strikes the ground or passes various media, powerful microwaves are emitted by coherent transition radiation (Fig. 1c). We further verify that this specific microwave in air plasmas naturally evolves into a microwave bubble. These results are demonstrated by particle-in-cell (PIC) simulation using the code JPIC^12^.

## Results

### Relativistic electron bunch

The assumption of isolated relativistic electron bunches in ball lightning events is based on high-energy phenomena^13^,^14^ discovered in cloud-to-ground lightning. A lightning flash^5^ starts with a negative leader propagating downward in a stepping process with each step tens of metres. This stepped leader has a corona 1–10 m in width. Moore *et al.*^15^ first detected >1 MeV radiation from a stepped leader. It was then observed that each step emits an x-ray burst^16^, which intensifies when the leader approaches the ground. Recent data^17^ shows that the last step or the so-called leader burst closest to the ground produces the strongest x-rays. Electrons accelerated by the stepped leader account for these detected x-rays, so that the electron acceleration is the most violent in the last step.

Friction force of electron motion in air is maximum at an energy of 100 eV, which defines a critical electric field *E*_*c*_ ≈ 30 MV/m^14^. Fields above *E*_*c*_ at the leader tip can accelerate thermal electrons to several keV^18^. This thermal runaway process^19^ can produce ~10^11^ electrons. The hot electrons can be further accelerated by the electric field between the leader tip and the ground, and serve as seed electrons to undergo avalanche in air^20^. The electron flux quickly rises as exp(*z*/*L*), where *L* is the avalanche length. The electron energy follows a Boltzmann distribution exp(−*k*_*e*_/7.3 MeV), such that the average energy is 7.3 MeV. Latest data analysis^21^ shows that collimating relativistic electrons are required to explain observed x-rays from the stepped leader, and should be either Boltzmann-distributed at 7 MeV or monoenergetic from 1 to 10 MeV.

The isolated x-ray bursts from the stepped leader are much shorter than 1 *μ*s^16^. On the other hand, metre-scale laboratory sparks in air^22^ can emit very similar x-rays as in natural lightning. Duration of x-ray bursts from laboratory sparks is generally sub-10 ns^23^ and can be as short as 1 ns^24^. Accelerated electrons are expected to have the same temporal structure as the x-rays from lightning or sparks.

**Figure 1:**
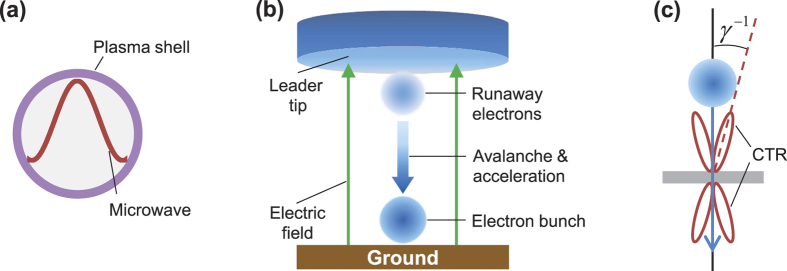
Ball lightning model. (**a**) Microwave bubble model. (**b**) Relativistic electron bunch generation. In the last leader step, a bunch of runaway electrons emerges from the leader tip, accelerates by electric fields between the leader and ground, and undergoes an avalanche. (**c**) Coherent transition radiation (CTR) of the electron bunch striking the ground or passing through aircraft skins. *γ* is the relativistic factor of electrons.

Accordingly, it can be expected that the last leader step generates a spatially well-defined relativistic electron bunch in a ball lightning event (see Fig. 1b). For simplicity, we assume that this bunch has a density profile *n*_*b*_ = *n*_*b*0_ exp(−*r*^2^/2*σ*^2^), where *n*_*b*0_ is the peak density, and *σ* is the characteristic radius. We take a bunch size (≃4*σ*) of tens of cm, i.e. ~1 ns in duration. As discussed later, a bunch with total electron number *N*_*b*_ = (2*π*)^3/2^*n*_*b*0_*σ*^3^ ≈ 10^14^ will lead to a microwave bubble. In the avalanche mechanism, this would need an avalanche path of 7*L*, corresponding to a multiplication rate of exp(7) ≈ 10^3^. The avalanche length *L* is 7–30 cm near the ground^14^ and should support the rapid amplification of these nanosecond bunches. Another mechanism^25^ predicts that the leader can directly generate ~10^16^ energetic electrons on a timescale of 1 ns without avalanche.

### Microwave generation

Transition radiation is generated from medium surfaces when an electron enters or emerges^26^ and can be coherent for an isolated electron bunch^27^. As the electron bunch reaches relativistic energies, its self-fields are predominantly transverse i.e. **E**_*b*_ ≃ *c***B**_*b*_^28^, which is very close to an electromagnetic wave. In this case, coherent transition radiation can be considered as the reflected wave of the bunch field from the medium surface^29^. Therefore, we can write the radiation energy as


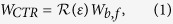


where 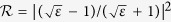 is the Fresnel reflection formula, *W*_*b*,*f*_ refers to the total bunch field energy, and *ε* is the medium permittivity. The radiation is strongest for a metal or perfect conductor where *ε* → ∞ and ℛ ≈ 1 in microwave region. In addition, a Boltzmann-distributed electron bunch turns out to produce almost the same transition radiation pulse as a monoenergetic one^30^.

**Figure 2:**
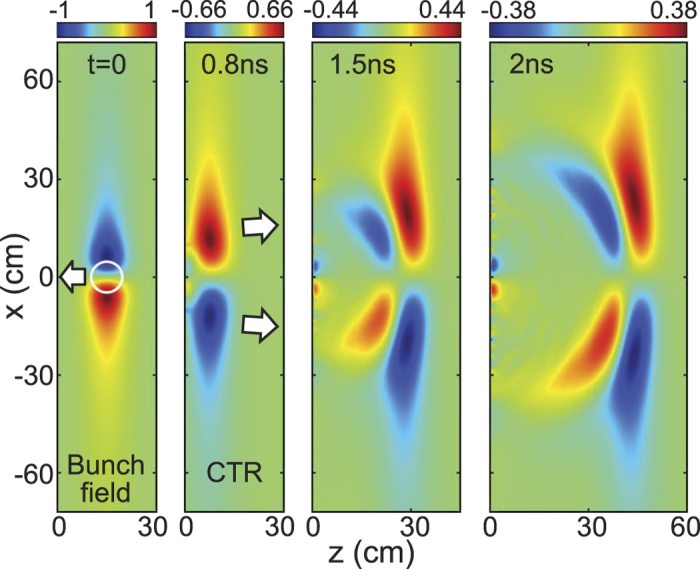
PIC results of microwave generation. Distribution of the initial bunch field and microwave fields at times 0.8 ns, 1.5 ns and 2 ns. The field is normalized to the bunch peak field *E*_*b*0_. In the leftmost panel, the bunch is left-going to the plasma surface at *z* = 0. The white circle marks the bunch region with a density of 0.5*n*_*b*0_. The radiation is a reflection of the bunch field and propagates along *z*. Arrows point to the field propagation direction. Parameters are given in the text and Methods.

The leftmost panel of Fig. 2 shows the transverse field *E*_*b*,*x*_ of a monoenergetic 7 MeV electron bunch with *σ* = 4 cm, which is normalized to the peak field 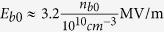. The bunch field is a unipolar wave with the same profile exp(−*z*^2^/2*σ*^2^) as the electron density along the direction of motion. Using JPIC^12^, we simulate the coherent transition radiation from a perfect conductor in Fig. 2. The radiation field *E*_*x*_ is initially opposite to *E*_*b*,*x*_ due to the conductor boundary, diffracts transversely, and quickly evolves into a bipolar pulse. This radiation has a central wavelength *λ* ≈ 7.5*σ* = 30 cm (i.e. 1 GHz). The rapid field evolution into the bipolar shape is due to diffraction losses of longer wavelength components in an unipolar pulse^31^. At normal incidence in Fig. 2, the radiation field is radially polarised with a ring-like intensity distribution. Oblique incidence^32^ can enhance the radiation production and lead to an asymmetric intensity pattern. Considering surface fluctuations and non-axisymmetric bunches, the actual radiation could contain only one high-intensity emission spot, which is linearly-polarised and will make bubble formation more easily.

### Microwave bubble formation

Laser solitons have been observed in both PIC simulations^33^,^34^ and experiments^35^,^36^,^37^ on relativistic laser-plasma interaction. The laser needs to exceed the relativistic field threshold *E*_*r*_ = *mcω*/*e*^38^ and is typically multi-cycle. The plasma is underdense with an initial density *n*_0_ < *n*_*c*_, where *n*_*c*_ = *ε*_0_*mω*^2^/*e*^2^ is the critical density^39^. During the laser propagation in the plasma, the self-phase modulation effect^40^ leads to a dramatic spectral broadening, which makes some part of laser energy to shift even below the background plasma frequency. Hence this part gets trapped in a plasma cavity with a half-cycle standing wave mode. The cavity is spherical and formed by evacuating electrons through the relativistic ponderomotive force^41^. The entire formation process takes tens of light cycles.

Here, we discuss the bubble formation for a mono-cycle microwave in Fig. 2. The microwave must get trapped within a few cycles before it is diffracted. In contrast to the mechanism discussed above, we find that the initial plasma must be overdense with *n*_0_ ≥ *n*_*c*_, where *n*_*c*_ ≈ 1.2 × 10^10^cm^−3^ at *ω*/2*π* = 1 GHz. The existence of such a bubble-formation regime for single-cycle waves indicates self-consistency of our theory. The collisional effect is included by embedding air friction^14^,^18^ into JPIC. We launch microwave pulses with wavelength *λ* = 30 cm into a uniform plasma. The simulation shows that the threshold field required for bubble formation is


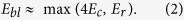


At 1 GHz, we have *E*_*r*_ ≈ 10.7 MV/m and *E*_*bl*_ ≈ 11*E*_*r*_ ≈ 120 MV/m, which is highly relativistic. Equation (2) clearly shows that the field needs to be greater than *E*_*c*_ to efficiently accelerate electrons, and reach the relativistic regime to completely expel electrons by the relativistic pondermotive force. Surprisingly, *E*_*r*_ matches with *E*_*c*_ to make the bubble formation possible. Here, we check the bunch parameters for giving the threshold field *E*_*bl*_. For the case in Fig. 2, we get *n*_*b*0_ ≈ 3.7 × 10^11^cm^−3^ and *N*_*b*_ ≈ 3.7 × 10^14^.

**Figure 3:**
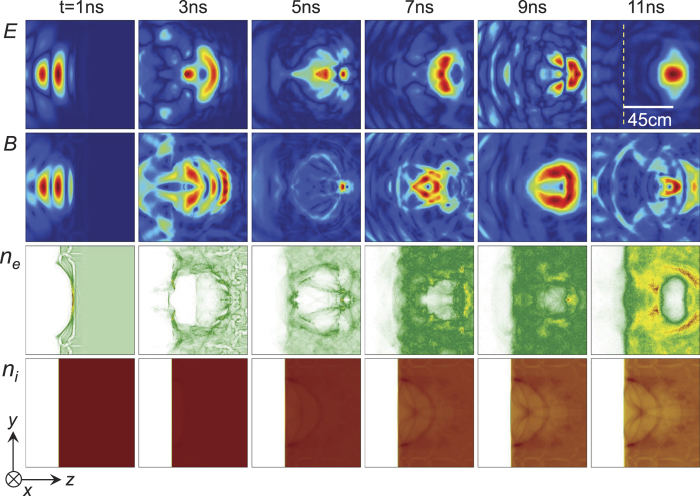
PIC results of microwave self-trapping and bubble formation. Snapshots of the microwave electric field *E* = |*E*_*x*_|, magnetic field 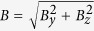, electron density *n*_*e*_, and ion density *n*_*i*_ from *t* = 1 ns to 11 ns. Vertical dashed line marks the plasma surface. Parameters are given in the text and Methods.

In Fig. 3, we take *n*_0_ = 4*n*_*c*_ and a microwave field of 310 MV/m, and let *t* = 0 when the field touches the plasma. Snapshots of microwave field and plasma density from *t* = 1 ns to 11 ns illustrate the entire process of microwave self-trapping and bubble formation. The radiation pressure of microwave first pushes electrons to pile up into a semicircular shell at *t* = 1 ns and leaves a low-density region at the rear. As the field is reflected by the front shell, peripheric electrons return to the low-density region and close up the cavity at *t* ≈ 3 ns. The field gets trapped and then evolves into a standing-wave mode. At *t* = 11 ns, a motionless electron cavity forms about 45 cm deep into the plasma, and then it becomes circular and keeps stable after *t* ≈ 15 ns. Meantime, heavy ions are slowly pulled out by the charge separation field.

**Figure 4:**
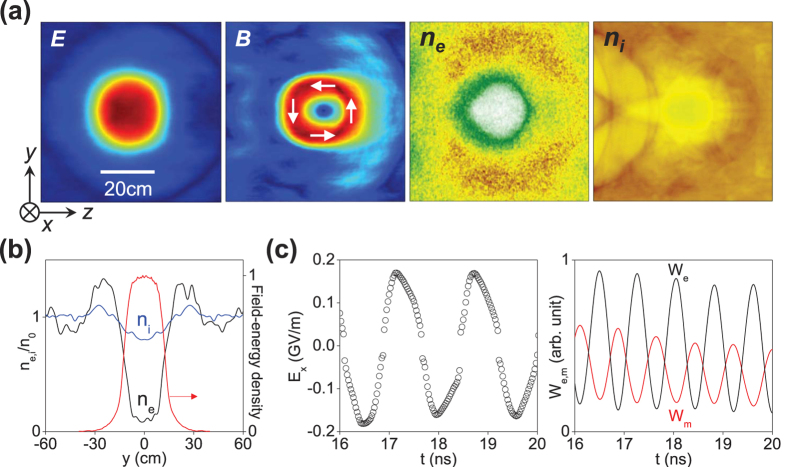
PIC results of stable microwave bubble. (**a**) Snapshots of the microwave electric field *E*, magnetic field *B*, electron density *n*_*e*_, and ion density *n*_*i*_ at *t* = 19 ns. White arrows mark the magnetic field direction. (**b**) Field energy density and plasma density *n*_*e*,*i*_ verses *y* across the bubble centre. (**c**) Evolution of the electric field, electric field energy *W*_*e*_, and magnetic field energy *W*_*m*_ in the bubble. Parameters are the same as Fig. 3.

In Fig. 4a,b, snapshots of the stable bubble at *t* = 19 ns show that the fields take on a half-cycle standing wave pattern, electrons have been almost emptied, and ions are partially evacuated. The electrostatic force between electrons and ions is balanced by the radiation pressure *ε*_0_*E*^2^/4 ≈ 64 kPa, where *E* = 170 MV/m is the standing wave amplitude. The periodic conversion between electric and magnetic energies in Fig. 4c confirms the standing wave mode. The confined field oscillates at a longer period of 1.6 ns. This redshift is caused by the Doppler effect and self-phase modulation. The cavity diameter is about 24 cm, half of the wavelength of the trapped field. For a ball shape, the confined field energy in Fig. 4b is about 800J. Tuning the microwave field, the trapped field energy in the bubble ranges from 200J to 1500J.

Three-dimensional field structure of microwave bubbles can be close to that of the light solitons observed in PIC simulation^34^. With energy loss of microwave by collisional absorption, the bubble is expected to convert into an electromagnetic cavity resonator. The fundamental mode at the lowest eigenfrequency in a spherical resonator^26^ is similar to that in a cylindrical cavity^28^, which resembles that shown in Fig. 4a.

### Explanation of the diverse properties

The properties of ball lightning^2^,^3^,^4^,^5^ are summarized from about 5000 published sighting reports.

#### Site of occurrence

As shown in Fig. 2, a planar surface is necessary for microwave generation at least with a size of ball lightning, which can be easily fulfilled in reality. Microwave emission is also affected by the ground reflectivity 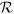. The soil permittivity *ε* increases with its moisture *m*_*s*_^42^. At 1 GHz, we get 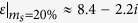 and 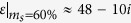, which correspond to 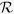 ≈ 25% and 56%, respectively. Rainfall can lead to *m*_*s*_ > 60%^43^ and thus is favorable for the ball lightning formation. As stated by Stenhoff^4^, more than 50% of reports show that medium or heavy rainfall happens before the observation. Moreover, there is 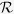 ≈ 65% for either pure or sea water^44^. Indeed, there are 18 reports at sea^2^ and a few reports over rivers^2^,^4^. Certainly, metal holds the highest chance of ball formation due to 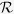 ≈ 1.

#### Relation to lightning channels

The lightning channel refers to the bright return stroke occurring after the stepped leader attaches with a positive leader rising from the ground. The starting place of this positive leader would be the lightning strike point. We show that ball lightning is caused by the stepped leader, which is invisible with the naked eye. The stepped leader and its mirror charge underground establish a dark channel for electron acceleration and avalanche. Obviously, the ball formation site is unrelated to the lightning strike point. Their separation should be within one step length of tens of metres typically. This successfully explains the reports where ball lightning does not form near the lightning channel or strike point^4^.

#### Appearance in aircraft

First, the avalanche electron energy 7.3 MeV is independent of the air density^13^, i.e. altitude. When lightning strikes an aircraft, the same bunch is presumably produced and enters the aircraft with an energy loss of ~2 MeV due to the ~0.6 cm aluminium skin^45^. Second, transition radiation^26^ is not sensitive to the energy of the relativistic electrons, and its efficiency from the electron emerging surface of the medium is almost the same as the reflection side discussed above. Therefore, the same intense microwave will arise inside the aircraft and form ball lightning there. In the same manner, ball lightning can appear in enclosed rooms.

#### Permeation through glass plates

Ball lightning is observed to enter rooms by passing through closed glass windows. In interference experiments of low-power microwave in metal cavity^46^, generated fireballs in air are observed to pass through a 3 mm ceramic plate intact. This is a direct result of the ability of microwave passage across dielectrics. The microwave bubble resembles a laser cavity. According to laser theory^47^, the internal standing wave will not be disturbed if a glass plate (~5 mm) is much thinner than the wavelength of microwave.

#### Shape

From dimensional analysis^12^, the microwave bubble of Fig. 4 in reality should be ball-shaped as its micrometre-scale counterpart in laser-plasma experiments^35^,^36^,^37^. The full trapping of the field in Fig. 2 can account for the 62 ring-shaped ball lightning reports^2^.

#### Size

Ball lightning has a common diameter of 20–50 cm^4^. Our theory shows that the diameter of microwave bubbles approximately equals the electron bunch length in the direction of motion. The bunch length of tens of cm is supported by x-ray duration measured from lightning and laboratory sparks, which can be as short as 1 ns.

#### Sound

Hissing, buzzing or fluttering sounds from ball lightning have been reported, which can be perfectly explained by the microwave hearing effect^48^,^49^. At 0.1 mJ/cm^2^, a microwave pulse (microsecond or shorter) at 0.2–3 GHz can induce an audible sound wave. The sound can only be heard by persons whose heads are irradiated by the microwave, and has been described as a hiss, buzz or knocking. Therefore, ball lightning can be silent during its lifetime. In Jennison’s sighting^50^, he was only 0.5 m from a cruising ball, and did not report any noise.

#### Spark

Ball lightning sometimes emits sparks, which can be caused by the ejection of charged particles along the electric field. Especially, the sparks are toward opposite directions in two reports^2^, which agrees with the linear polarisation of standing wave in the bubble.

#### Spectrum

Recently, Cen *et al.*^51^ recorded an optical spectrum of ball lightning. The spectrum contains emission lines of atoms in air and soil. Interestingly, the spectral intensities of O and N atoms oscillate at 100 Hz, twice the frequency of the adjacent power lines (35 kV, 50 Hz). The latter is only 20 m from the ball and can produce a 50 Hz electric field of ~1 V/cm^52^ at the ball. This field can induce electron drift on the ball surface by tens cm (see Methods). This drift motion can perturb the spectral emission in the plasma shell. The spectral intensity should be independent of drift direction and varies at 100 Hz. The ball is attached to the soil on a hillside, where electrons cannot feel the oscillating field due to the screening effect. Thus, Si, Fe and Ca in soil glow steadily^51^.

#### Odour

Ionized air can produce O_3_ and NO_2_^5^,^53^, both of which have an acrid smell.

#### Decay

The microwave bubble decays silently once the internal radiation is exhausted. When it is strongly disturbed or pierced by a conductor, the leaking radiation can launch a shock wave like an explosion.

#### Injury and damage

Most reported injuries and damages can readily be attributed to ordinary lightning^2^,^4^. However, Stenhoff^4^ noticed that some superficial burns are difficult to explain. In the Smethwick event^4^,^54^, the female witness did not get an electric shock but felt a burning heat all over. Wooding^55^ estimated that she received 250J whole-body ionizing radiation, which can be due to the electrons from the stepped leader and also be responsible for the redness on her hand and legs. She heard a knocking-like sound (rattle) from the microwave hearing effect. Her legs were numbed, which can be due to nerve damage by the microwave at 0.1J/cm^2 ^56. When she brushed the ball away with her hand, the ring was burning into her finger. Wooding calculated that this rapid heating would need a resonant microwave at 1 GHz with an field of ~1 MV/m, which agrees well with our model. Others^57^ reported skin redness, vomiting and loss of hair, which are typical results of ionizing radiation^58^. As reported by X. Zhang and Q. Yan in Shanxi Daily (8 Aug. 2014), during a thunderstorm on 5 Aug. 2014, a red ball of fire 40 cm in diameter was witnessed entering an office through an open window at the local Water Conservancy Bureau in Xinjiang, Shanxi, China. The ball lasted for less than one second and then exploded loudly. Five computers in the room were damaged, which is a direct result of high-power microwaves^56^.

#### Motion

Near the ground, ball lightning moves mostly horizontally at about 2 m/s^2^ and usually travels with the wind^3^. A light breeze typically at 1.5–3 m/s^59^ can account for this motion speed. However, air convection will raise the ball if the background air is heated up by the ionized plasmas. Assuming a constant heat power of 100 W, we obtain a convection speed 23 cm/s for the ball of size 30 cm (see Methods). Thus, the upward motion is not notable compared with the horizontal motion. Several models^2^,^4^ speculate that the ball could take a positive charge due to the greater mobility of electrons compared with ions. The charged ball can further resist the buoyancy or air convection by an attractive force from its mirror charge underground. Moreover, like a charged particle self-accelerating into an open waveguide^60^, the ball can enter rooms through chimneys.

#### Lifetime

The typical lifetime of ball lightning is 1–5 seconds. Statistical analysis^61^ shows that increase in humidity decreases the lifetime of the ball, which can be due to microwave absorption by vapour. Experiments^62^ show that fireballs in air produced by a 5 kW, 2.45 GHz microwave can last for ~0.5 s after the source is turned off. Our self-organized microwave bubble can have the same potential to persist for a scale of seconds. Zheng^11^ calculated that hundreds of joule microwaves can maintain the plasma shell of the bubble for a few seconds. Air plasmas continuously depleted by recombination are refilled by microwave heating. Non-neutral plasmas shown in Fig. 4b can further resists the recombination loss.

## Discussion

Experiments are required to verify our theory. First, forming a microwave bubble in laboratory will need hundreds of gigawatt microwave, which is one order of magnitude higher than the manmade sources. As stated in ref. 56, it is technically feasible to enhance current microwave devices to 100 GW. Alternatively, one can adopt a high-power electron beam^63^ to directly simulate the mechanism proposed in Fig. 1. Second, on the lightning research, we suggest to detect microwave radiation at GHz near a lightning strike point. We already show that trans-ionospheric pulse pairs from lightning are caused by the same radiation mechanism^64^, which supplies a physical evidence of our theory. On attempts to create ball lightning by rocket-triggered lightning^65^, we propose to use ungrounded wires^5^ rather than grounded ones because ball lightning is thought to be only related to the stepped leader. Perhaps intense lasers can trigger lightnings by producing an ungrounded plasma channel near thunderclouds^66^. For *in situ* investigation of ball lightning, we suggest to look for evidence of high-flux energetic electrons. Finally, we note that relativistic terahertz waves could be produced from laser-accelerated hot electrons emerging from solid foils by coherent transition radiation^67^ or laser-driven plasma waves in gas target^68^. In particular, the former scenario is very close to the scheme in Fig. 1 and may lead to a millimetre-scale terahertz radiation bubble.

## Conclusion

In conclusion, based on a reasonable assumption on the electron bunch, we have constructed a self-consistent theory on the microwave generation and ball lightning formation. The theory successfully explains many properties of ball lightning. For the first time, we revel that ball lightning is an alarm signal of the existence of ultrastrong microwaves and abundantly hazardous electrons near the ground or aircraft. This result is of great significance for lightning protection and aviation safety. Moreover, it is hoped that our work will stimulate research activities in relativistic microwave physics and technology, an unexplored area before.

## Methods

### Dimensional analysis

The interaction of relativistic electromagnetic wave and collisionless plasmas is governed by the Maxwell’s equations and relativistic Lorentz equation of electrons and ions. When time and space are normalized to the cycle and wavelength of electromagnetic wave respectively, the whole system only depends on two dimensionless quantities 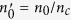 and 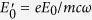, where *n*_0_ is the initial plasma density, *n*_*c*_ = *ε*_0_*mω*^2^/*e*^2^ is the critical density, *E*_0_ is the initial field amplitude, *e* is the fundamental charge, *m* is the electron mass, *c* is the light speed, *ω* is the angular frequency, and *ε*_0_ is the vacuum permittivity. If 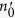 and 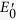 are same for any systems with different wavelengths *λ* = 2*πc*/*ω*, the physical process should be identical in these systems^12^. By the way, 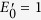 defines the relativistic field threshold *E*_*r*_ = *mcω*/*e*. For a microwave at *λ* = 30 *cm*, we have *E*_*r*_ = 10.7 MV/m (*I*_*r*_ = 1.5 × 10^7^W/cm^2^) and *n*_*c*_ = 1.2 × 10^10^cm^−3^.

### PIC simulation

All simulations are done with the JPIC code^12^, which self-consistently solves the Maxwell’s equations and relativistic Lorentz equations for electrons and ions in a two-dimensional space. JPIC applies a field solver free of numerical dispersion in the propagation axis and can accurately simulate the dynamics of half-cycle electromagnetic waves^31^. The simulation of transition radiation in Fig. 2 is performed in the *xz* plane. An overdense plasma is used to represent the conductor and its density has a negligible effect on the results. In Fig. 2, we take a density *n*_0_ = 50*n*_*c*_ and resolution of 100 and 80 grids per wavelength (*λ* = 30 cm) along the *z*- and *x*-axes, respectively. The simulation of bubble formation in Figs 3 and 4 is done in the *yz*-plane. Since the collision frequency in air is ~10^12^Hz, i.e., thousands of collisions per cycle, which makes the resolution of individual collisions unrealistic in the present work. For the simulation, we embed the effective air friction force within an electron energy range [1 eV, 1 GeV]^14^,^18^ into the Lorentz equation of electrons. The microwave field *E*_*x*_ is perpendicular to the simulation plane and propagates from a vacuum to a uniform plasma along the *z*-axis. The microwave pulse has the form 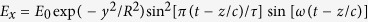, where *E*_0_ = 310 MV/m is the field amplitude, *R* = 9 cm is the spot size, *τ* = 2 ns is the duration, and *ω*/2*π* = 1 GHz is the central frequency. The full width at half maximum of the field envelope is *τ*/2 = 1 ns. There are 80 and 64 grids per wavelength along the *z* and *y* axes respectively. Air molecules take an average molecular weight 28.97 and charge state *Z* = 1. In Fig. 3, to clearly recognize the bubble structure, color bars are based on specific values at each moment, and therefore no quantitative relation exists among the different panels.

### Microwave effects on humans

Microwave can penetrate deeply into the tissue and cause an influence by thermal effects. Microwave hearing^48^,^49^,^56^ is the lowest power effect on humans and occurs when the absorbed energy in the brain tissue reaches 10 *μ*J/g for a 10 *μ*s pulse. For a typical adult brain with 14 cm in diameter and 1.4 kg in weight, we get an energy flux threshold of 0.1 mJ/cm^2^. Experiments^48^,^49^ show this hearing effect induced by 0.2–3 GHz microwave pulses with 1−100 *μ*s in duration. Theoretical analysis reveals that rapid (~*μ*s) temperature rise (~10^−6^ degree) leads to a thermoelastic expansion of tissue, which launches an acoustic wave travelling by the skull to the inner ear. The audio frequency is located at an audible high-frequency band of 7–15 kHz, which is responsible for the sounds of hiss, buzz, knocking or clicking^48^. Although rather resistant to ionizing radiation^69^, sensory nerves in the peripheral nervous system are found to be particularly sensitive to the microwave^70^. Occurring at 0.1 J/cm^2 ^56, nerve damage can lead to a numbness in the limbs^71^. In our theory, the microwave reaches ~1 J/cm^2^ for the ball formation, which is enough to induce both microwave hearing and nerve damage on witnesses.

### Electron drift in air

In an electric field *E*_*f*_ sin(2*πf t*) with frequency *f*, electrons in air gain a drift velocity *V*_*e*_ = *μ*_*e*_*E*_*f *_sin(2*πft*)^13^, where *μ*_*e*_ is the electron mobility. The amplitude of electron drift is then 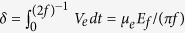. Taking *E*_*f*_ = 1 V/cm, *f* = 50 Hz and *μ*_*e*_ = 0.6 m^2^/V/s^18^, we have *δ* ≈ 38 cm. Ions have *δ* ≈ 0.1 mm due to its small mobility.

### Air convection

The microwave bubble will heat up the initially uniform air by electron-molecule collisions. When the temperature rises, air will expand and be lifted up by buoyancy, which leads to air convection. Assuming the temperature change Δ*T* is small, the convection speed is given by 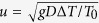
72, where *g* = 9.8 m/s^2^ is the gravitational acceleration, *D* is the bubble size and *T*_0_ is the initial air temperature. If thermal energy is transferred primarily by the air convection, one has *H* = *C*_*p*_*ρ*_0_*Su*Δ*T*, where *H* is the total heat power of bubble, *C*_*p*_ is the specific heat capacity of air, *ρ*_0_ is the air density, and *S* = *π*(*D*/2)^2^ is the cross sectional area of bubble. From these relations, we obtain


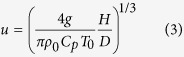



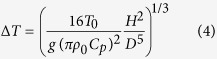


At the room temperature *T*_0_ = 293 K, we have *ρ*_0_ = 1.2 kg/m^3^ and *C*_*p*_ = 1 × 10^3^J/K/kg^73^. For a bubble with *D* = 30 cm and *H* = 100 W, the convection speed is *u* ≈ 23 cm/s and temperature increase is Δ*T* ≈ 5 K. We also get the Reynolds, Peclet, and Rayleigh numbers of this system as 4.5 × 10^3^, 3.2 × 10^3^, and 1.4 × 10^7^ respectively. These dimensionless numbers confirm that convection is the dominant mechanism of heat transport^72^.

## Additional Information

**How to cite this article**: Wu, H.-C. Relativistic-microwave theory of ball lightning. *Sci. Rep.* **6**, 28263; doi: 10.1038/srep28263 (2016).
